# How digital technology can contribute to timely and effective recognition and response to opioid overdose events

**DOI:** 10.1192/j.eurpsy.2024.58

**Published:** 2024-08-27

**Authors:** A. M. Baldacchino

**Affiliations:** Medicine, St Andrews University, St Andrews, United Kingdom

## Abstract

**Objectives:**

To discuss novel approaches in the development early detection, response and interventions of drug overdoses.

**Rationale:**

There is an urgent need to research and develop novel strategies to rapidly and accurately detect, respond, and treat them with the ultimate goal of reducing drug deaths secondary to fatal drug overdose incidents. This should be additional to supporting communities and networks able to intervene utilizing novel public health approaches.

**Methods:**

We will describe technologies and associated systems that are able to accelerate detection and result in a timely response to potential overdose with effective and timely intervention to these occurrences using digital technologies and therapeutics. This will be contextualised around novel public health approaches.

**Results:**

We will describe 11 protypes as part of a £5 million UK inititiative. The themes will include*:*

1. Use of discrete digital technology for easy use by people who use drugs in clinical and non-clinical settings
2. Simple alert / responder pathways that created effective responses to potentially fatal overdose events
3. Enhance innovative therapeutics as antidotes to overdose episodes
4. Novel public health approaches

**Conclusion:**

The use of remote monitoring devices like wearables and smartphone applications, paired with artificial intelligence and innovative therapeutics is an emerging field of research. This needs to be balanced around novel public health approaches.

**Disclosure of Interest:**

None Declared

